# Semantic annotation of Glycomics and Glycoproteomics methods

**DOI:** 10.1093/glycob/cwaf072

**Published:** 2025-10-31

**Authors:** Wenjun Wang, Valeriia Kuzyk, Guinevere S M Lageveen-Kammeijer, Magnus Palmblad

**Affiliations:** Center for Proteomics and Metabolomics, Leiden University Medical Center, Postbus 9600, Leiden, RC 2300, The Netherlands; Department of Bioanalytical Chemistry, AIMMS: Amsterdam Institute of Molecular and Life Sciences, Vrije Universiteit Amsterdam, PO Box 7161, Amsterdam MC 1007, The Netherlands; Analytical Biochemistry - Groningen Research Institute of Pharmacy, University of Groningen, Antonius Deusinglaan 1, Groningen, AV 9713, The Netherlands; Center for Proteomics and Metabolomics, Leiden University Medical Center, Postbus 9600, Leiden, RC 2300, The Netherlands

**Keywords:** glycomics, glycoproteomics, ontologies, text mining

## Abstract

Glycomics and glycoproteomics represent the systematic exploration of glycan structures and glycoprotein compositions within biological systems, aiming to elucidate their roles in physiological and pathological processes, including cancer, inflammation and infectious diseases. To support this investigation, glycomics and glycoproteomics utilize a diverse array of methodologies from molecular biology, biochemistry, analytical chemistry and bioinformatics. In this study, we investigated the semantic representation experimental workflows in glycomics and glycoproteomics publications through graph-based annotation using combination of existing domain-relevant ontologies. Rather than adhering to evolving metadata standards, this investigation explored a broad spectrum of biomedical and analytical ontologies to identify optimal annotations for the generative (e.g. sample preparation and derivatization) and transformative (e.g. separation and detection) phases of the workflow. The results show that integrating several ontologies yields more precise annotations than relying on a single one. However, several challenges arose, particularly where methodological reporting lacked critical metadata, such as derivatization conditions or glycan release protocols. Furthermore, the annotations imply that methodologies in the glycomic and glycoproteomic fields are more complex, on average, than those in other scientific fields. The results suggests that, while some specific concepts are missing in the ontologies, a limited number of ontologies adequately encompass the majority of aspects related to glycomics and glycoproteomics experiments. These can serve as a foundation for community-wide metadata standards and direct future efforts to refine and expand the ontologies for glycoscience research.

## Introduction

Glycoproteins play a vital role in almost every biological process, influencing functions from cell signaling to immune responses (Rabinovich et al. 2012). The complexity of glycans, ranging from their diverse structural configurations to their vast quantitative dynamic range, presents unique challenges for researchers. To fully understand these molecules, a multifaceted approach is required, one that combines biochemistry, analytical chemistry and emerging fields like machine learning and artificial intelligence. Several open-access resources to describe step-by-step experimental methods, like Nature Protocols (Glycoscience) (Kaji et al. 2006; Wang et al. 2011; Kim et al. 2012; Kolarich et al. 2012; Falck and Wuhrer 2024), The Essentials of Glycobiology (Protocol included) (Varki et al. 2022) and Glycoscience Protocols (GLYcoPODv2) (Nishihara et al. 2021), have been extensively developed in the glycoprotein field. Each experimental step, from enzyme-based cleavage and separation techniques to ionization methods, can impact the resulting data in significant ways. Variations in sample recovery, glycan stability or derivatization effects can complicate comparisons between datasets from different studies or laboratories. This highlights the need for highly detailed metadata that captures these experimental nuances, providing essential context for accurate data interpretation. For the field of glyco(proteo)mics to progress, embracing the FAIR (Findable, Accessible, Interoperable, and Reusable) principles is essential (Wilkinson et al. 2016). This means not only generating robust and high-quality datasets but also ensuring the data is easily accessible and comparable. Several key repositories, like PRIDE (Perez-Riverol et al. 2022) and GlycoPOST (Watanabe et al. 2021) for mass spectrometry (MS) data, the UniCarb-DR (Rojas-Macias et al. 2019) repository and UniCarb-DB (Hayes et al. 2011) curated database of mass spectra and glycan structures, and the GlyTouCan (Tiemeyer et al. 2017) glycan structure repository, have become central to housing glyco (proteo) mics data, with efforts such as SNFG (Varki et al. 2015) providing a standard nomenclature for spectrum annotation and glycan identification. However, for these datasets to be truly interoperable, they must be accompanied by comprehensive metadata, including detailed descriptions of experimental protocols. This metadata, formalized through controlled vocabularies and ontologies, ensures that data can be reused, integrated effectively, and are machine readable. Importantly, the experimental steps used to generate the data, including enzymatic digestion, separation and ionization influence the information content of the data found in repositories or presented in papers in important ways, directly impacting the quality and comparability of the data. Ontologies play a critical role in this process, offering standardized frameworks that allow for consistent representation of experimental methods across different studies. Several hundred ontologies, many of which can be found in the Open Biological and Biomedical Ontology (OBO) Foundry (Smith et al. 2007), are used in the life sciences, each covering a different domain in which they are designed to model knowledge. These domains can be broad or narrower, and there are often overlaps between ontologies, as also demonstrated here. While existing and maintained ontologies like the Ontology for Biomedical Investigations (OBI) (Bandrowski et al. 2016), the Chemical Methods Ontology (CHMO) (Pachl et al. 2020), and the Ontology of bioscientific data analysis and data management (EDAM) (Black et al. 2022) provide a strong foundation, gaps remain, particularly when it comes to unique methods and analyses of the fields of glyco (proteo)mics. The importance of this process was highlighted by the EACH100 (Excellence in Analytical Chemistry) study by Palmblad et al. (Palmblad et al. 2022), whose study used semantic graphs to annotate 100 esearch papers involving MS in the journal *Analytical Chemistry*. This study explored the fit-for-purpose of ontologies in representing experimental methods and assessed the potential for natural language processing (NLP) methods (Young et al. 2018), including large language models (Simon et al. 2024) to extract meaningful metadata from scientific papers. While NLP has made great strides in understanding the general text, technical scientific language presents its unique challenges. In particular, STEM literature, especially in niche areas like glyco(proteo)mics, does not fit neatly into the existing training models. The training data pool is insufficient and existing texts are often incomplete, omitting trivial to domain experts parts, which NLP models cannot contextualize and restore. Moreover, tokenization and technical instructions are sequential/directional, as well as lexically different in such texts. Tailoring of NLP into Technical Language Processing (TLP), such as in the INDRA assembler (Bachman et al. 2023) which integrates NLP and curated databases to assemble causal graphs, relies on a structured and expertly annotated domain knowledge base and representative, domain-specific, examples that we aim to provide with this work. In this study, we take a similar approach as in the preceding study by examining which ontologies effectively capture the distinct phases of glyco(proteo)mics experiments. In contrast to the previous study, which focused on a single journal (offering a consistent structure for NLP evaluation), we expand the scope to encompass more diverse reporting styles and experimental descriptions. The expansion aims to reduce biases caused by journal-specific editorial policies and open access publishing agreements, thereby enhancing the robustness of our semantic annotation and NLP evaluation. While EACH100 used 100 publications in Analytical Chemistry, including several publications from the same research groups, our approach prioritizes diversity over volume. Specifically, we curated a corpus of 20 recent papers, each from a different journal and authored by a different research group. a wide range of experimental techniques, from enzymatic workflows, capillary electrophoresis (CE), and liquid chromatography (LC), to MS and nuclear magnetic resonance (NMR). The research topics span diverse biological domains such as cancer, neurodegenerative disease, parasitology and food science. This study complements existing standardization efforts in the field of glycomics, particularly the MIRAGE (Minimum Information Required for A Glycomics Experiment) guidelines (https://www.beilsteininstitut.de/en/projects/mirage/), as well as related initiatives (Taylor et al. 2007; Deutsch et al. 2023) aimed at improving data reporting and interoperability in glycoscience. While MIRAGE adopts a prescriptive framework that defines required reporting elements, our research takes a descriptive approach, ontologies in the Ontology Lookup Service (Côté et al. 2008) semantically represent the different phases of glyco(proteo)mics experiments. By annotating 20 recent publications drawn from a diverse range of journals and research groups, we aim not only to improve data standardization but also to illuminate the ways experimental techniques are used in tandem or sequence. For example, workflows that incorporate PNGase F digestion before LC–MS analysis reveal characteristic methodological sequences that can be captured through semantic annotation. This approach helps clarify how analytical steps interconnect and evolve within glyco(proteo)mics workflows. Ultimately, by integrating experimental methods with structured ontological frameworks, we move toward a more universal understanding of how to design, report, and interpret glyco(proteo)mics experiments. Improved standardization and metadate richness will pave the way for better data integration, cross-study comparisons, and facilitate the reproducibility of complex glyco-analytical workflows. These advances will foster deeper insights into how glycans and glycoproteins drive biological processes and contribute to diseases, strengthening the foundations of biomedical research and therapeutic innovation.

## Results

The 20 annotated papers are listed in Table 1. When selecting papers for annotation, the most restrictive parameters were the requirements that only open access papers could be used (for text accessibility), the papers should be recently published (initially considering publications from 2020–2024), and published in different journals. Those papers, nevertheless, represent a snapshot of the recent scientific literature in the fields of glycomics and glycoproteomics, and describe analyses of *O*- and *N*-glycosylation, as well as both types of glycosylation in the same study. In total, we annotated 470 experimental procedures as nodes with 580 inputs and outputs as edges using 22 ontologies for the annotation of the nodes and 16 for the edges. These edges represent either a physical material such as a sample or reagent, or data generated through a measurement, for example by MS or NMR. Additional information on the annotated papers and ontologies is included in the Table S1.

**Table 1:** The corpus of 20 annotated open-access glycomics and glycoproteomics papers published in 20 different journals by 20 individual research groups between 2021 and 2023.

First author	Main topic	*O*- or *N*-linked	Fig.	Year	Journal (publisher)	PMID
Duivelshof et al. 2021	Glycomics	*N*	TOC	2021	*Pharmaceutics* (MDPI)	34834160
Hargett et al. 2021	Glycoproteomics	*N*	-	2021	*Molecules* (MDPI)	34299586
Liew et al. 2021	Glycomics	*N*	-	2021	*Commun. Chem.* (Springer Nature)	36697781
Madunić et al. 2021	Glycomics	*O*	Supp.	2021	*Cell. Mol. Life Sci.* (Springer)	32236654
Ret et al. 2022	Glycomics	*N*	1	2021	*Talanta* (Elsevier)${\kern0em }^i$	35193013
Wilkinson et al. 2021	Glycomics	*O*	1	2021	*J. Proteome Res.* (ACS)	34191522
Alvarez et al. 2022	Glycomics, Glycoproteomics	*N*	-	2022	*ACS Omega* (ACS)	36385894
Chia et al. 2022	Glycomics	Both	1	2022	*Foods* (MDPI)	35804766
Cao et al. 2022	Glycoproteomics	*N*	1	2022	*Nat. Commun* (Springer Nature)	35798744
Luo et al. 2022	Glycoproteomics	*N*	1	2022	*Front. Immunol.* (Frontiers)	36189210
Petralia et al. 2022	Glycomics	*N*	-	2022	*Sci. Rep.* (Springer Nature)	36131114
Polasky et al. 2022	Glycoproteomics, Bioinformatics	*N*	TOC	2022	*Mol. Cell. Prot.* (Elsevier)	35091091
De Haan et al. 2022	Glycomics	Both	1	2023	*Anal. Chem.* (ACS)	35245040
Dutt et al. 2023	Glycoproteomics	*N*	1	2023	*Prot. Clin. App.* (Wiley)	37147936
Liu et al. 2023	Glycoproteomics	*N*	1	2023	*Exp. Ther. Med.* (Spandidos)	37753295
Lohia et al. 2023	Glycoproteomics	*O*	1	2023	*Int. J. Mol. Sci.* (MDPI)	36982475
Parsons et al. 2023	Glycoproteomics	*N*	-	2023	*Front. Mol. Biosci.* (Frontiers)	37383151
Takakura et al. 2023	Glycoproteomics	*O*	1	2023	*Front. Oncol.* (Frontiers)	36845686
Zhou et al. 2023	Glycoproteomics	*N*	TOC	2023	*Anal. Bioanal. Chem.* (Springer)	36607393
White et al. 2024	Glycoproteomics	*Both*	2a	2024	*Nat. Biomed. Eng.* (Springer Nature)	37474612

${\kern0em }^i$ This paper is open access, but not in PubMed Central and hence excluded from the analysis of the correlation between the numbers of nodes and words, and Fig. 2B.

The figure refers to an illustration in the graphical abstract/table of contents graphic (TOC) or a figure in the paper describing part or all of the experimental workflow. As can be seen from the table, this is now a fairly common practice, with the majority (70%) of the papers having at least some human-readable graph representation of the method in the paper or the associated TOC graphic.

### Example of a semantically annotated method

**Fig. 1:**
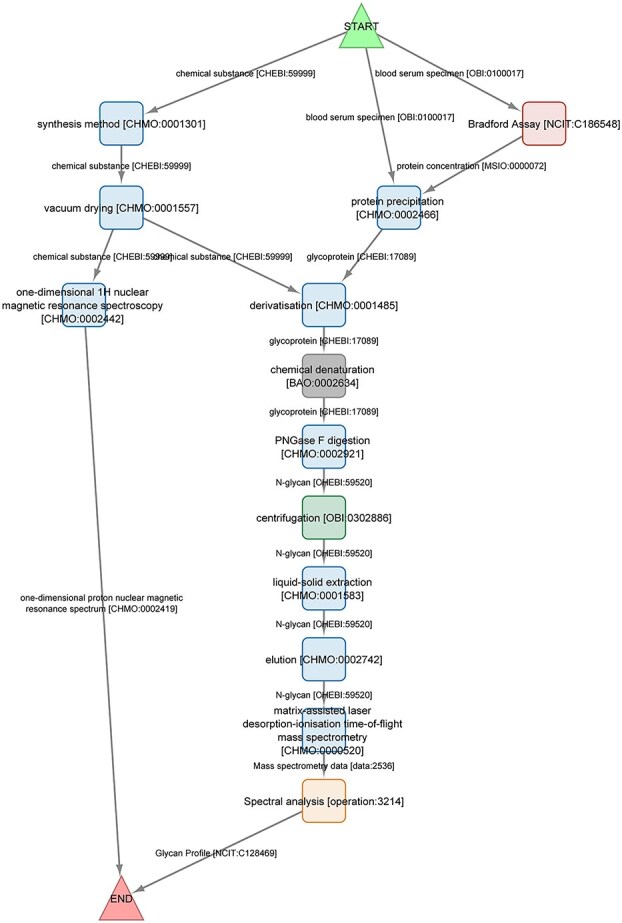
Semantic annotation of the experimental section in the paper by Davide Ret and co-workers on a DMTMM-mediated methylamidation method for MALDI-MS analysis of *N*-glycans in biological fluids. While most steps in the method are general, some are more glycomics-specific, such as PNGase F digestion [CHMO:0002921]. The method was demonstrated on both blood serum and intestinal lavage. However, the latter was not found in any of the searched ontologies. The term “lavage” in OBI [OBI:0600044] and NCIT [NCIT:C38068] refers to the *process* rather than the material, though the general term “lavage fluid” is defined in NCIT [NCIT:C103411]. The first figure in the paper also describes the glycan preparation part of the method.

An example of an annotated methods section is illustrated in Fig. 1, which includes several glycomics specific experimental steps. These steps are semantically annotated using multiple ontologies, including the BioAssay Ontology BAO, CHMO, EDAM, the National Cancer Institute Thesaurus (NCIT) and OBI. Among these, CHMO plays a particularly prominent role in capturing the chemical and procedural aspects of the workflow. When referring to terms from the ontologies, we use the spelling and capitalization from the ontologies. The annotated method starts with *chemical substance(s)* [CHEBI:59999] and *blood serum specimen(s)* [OBI:0100017] and describes all major transformative or generative steps, ending with a *Glycan Profile* [NCIT:C128469] and NMR spectrum, specifically a *one-dimensional proton NMR spectrum* [CHMO:0002419] demonstrating the quality of the synthesized reagents. The nodes correspond roughly to the paragraphs in the methods section of the article. Annotation.

### Complexity and corpus comparison

**Fig. 2:**
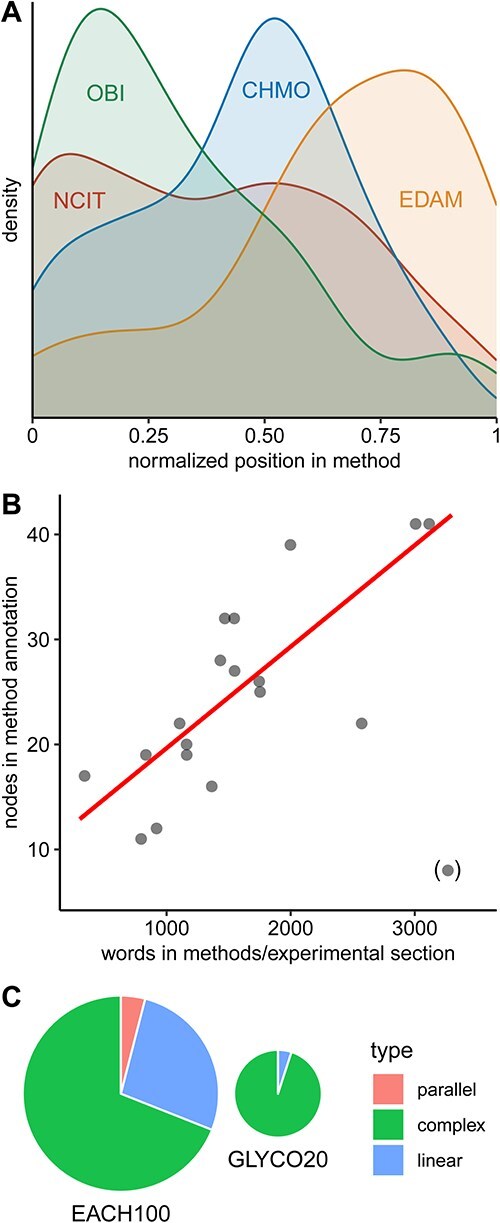
The distribution of the most frequently selected ontologies A) shows that NCIT has the broadest coverage, from sampling to the data analysis. However, by combining several ontologies, such as OBI, CHMO and EDAM, we can cover the same range and often find more precise terms than those in NCIT. Where OBI covers steps related to sampling biological systems or getting samples from a biobank, CHMO can be used to describe the analyses of those samples and EDAM the analysis of the resulting data. There is some overlap between all ontologies, most between OBI and NCIT and least between OBI and EDAM. The correlation between the number of words and the number of nodes in the annotations B) is stronger than in the EACH100 annotations, suggesting that the journal and possibly different journal requirements have less impact than the type of experiments described. Even if all 100 experiments annotated in EACH100 involve MS, they cover a very wide range of applications. The GLYCO20 set, though spanning 20 different journals, is all on glycomics or glycoproteomics experiments (most also using MS). This is also reflected in the distribution of the types of methods C), with all but one method in GLYCO20 being “complex,” with multiple branches but no single parallelized step, as in a comparison of many methods.

All 20 annotations are available in the Graph Modelling Language (GML) on GitHub (https://github.com/magnuspalmblad/GLYCO). The distribution of the most frequently used ontologies are illustrated in Fig. 2A, demonstrating that the CHMO ontology has good coverage of the analytical chemistry methods, which are often central in glyco(proteo)mics workflows. The more biological ontologies like OBI are useful to describe the biological system and its sampling defined at the beginning of methods, and the dedicated bioinformatics ontology EDAM is preferred in the annotation of the data analysis and visualization. The average length of the 20 methods section in the corpus here is 1548 words, 44% more than the average 1078 words in the previously annotated *Analytical Chemistry* methods sections (Palmblad et al. 2022). The GLYCO20 annotations have on average 23 nodes (not including the start and end nodes) and 29 edges (Table S2), compared to 12 nodes and 15 edges in the EACH100 annotations. The number of nodes generally correlates with the length of the method section (Fig. 2B). In accordance with previous observations (Palmblad et al. 2022), there is also great variability in writing styles. Moreover, nearly all of the annotated papers in the GLYCO20 dataset exhibit complex, branched workflows, with only a single paper following a linear experimental structure (Fig. 2C). This contrasts sharply with the EACH100 dataset, where a larger proportion of the workflows were linear, and several publications featured a parallel structure.

### Corpus diversity and annotation precision

Compared to the previous study that looked only at papers in the ACS journal *Analytical Chemistry*, the papers were selected from a larger and more geographically representative corpus. Of the 2756 open access articles published between 2020 January 1 and 2024 December 31, 580 corresponding authors have affiliations in the United States, 419 in China, 240 in Australia, 152 in Canada, and 142 in the Netherlands. While the NCIT ontology (or thesaurus) in principle covers all aspects of the annotated experiments (from sample to statistical analysis), precision would be lost if having to use, for example, the generic *Capillary Electrophoresis* [NCIT:C17637] (a method often used to separate released glycans) rather than the specific type of CE. In CHMO, there are four subclasses of *capillary electrophoresis* [CHMO:0001024] (*capillary affinity electrophoresis*, *capillary isoelectric focusing*, *capillary isotachophoresis*, and *capillary sieving electrophoresis*) and one sub-subclass (*capillary gel electrophoresis*, subclass of *capillary sieving electrophoresis*). In our annotations, we sought to use the most precise term applicable to describe the experimental methodology. Another choice that could be harmonized in future annotation efforts is the preference for several primitive or one combined term when both options are available. These combined terms are particularly well represented in CHMO, which includes many hyphenated techniques, such as *liquid chromatography–tandem mass spectrometry* [CHMO:0000701] that could also be annotated using more primitive terms for *liquid chromatography* [CHMO:0001004] and *mass spectrometry* [CHMO:0000470] without loss of information. In some cases, new or less common hyphenations such as *hydrophilic interaction chromatography* [CHMO:0002262] or *graphitized carbon liquid chromatography* [CHMO:0002924] in combination with *tandem mass spectrometry* [CHMO:0000575] need to be described using the individual techniques. For consistency, annotators may lean toward annotating all methods this way, even when the methods are included as hyphenated combinations. This may also be assisted through existing additional relationships in the ontology, such as the “has proper occurrent part” from *liquid chromatography-mass spectrometry* to *liquid chromatography*.

## Discussion

### Challenges in ontology coverage and term selection

The annotators’ background in glyco(proteo)mics experience of the annotators aided in the effort through an understanding of the types of experiments common in the field and an awareness or appreciation of minor significantly contributed to the annotation process by providing insights into commonly used experimental workflows and enabling recognition of subtle but important procedural details. Notably, some experimental concepts and terms appear across multiple ontologies, which can lead to redundancy or ambiguity in annotation. For example, *solid phase extraction (SPE)* is a widely used technique in glycomics for analyte enrichment or purification, definitioned in several ontologies. The CHMO [CHMO:0001583] entry is imported into four other ontologies (CAO, MICRO, PRIDE, and PROCO), all of which are interoperable through ontology mapping. However, the same concept is defined independently in OMIT [OMIT:0025161], despite OMIT being originally developed for data exchange in the microRNA domain. This overlap underscores the importance of careful term selection and ontology alignment to ensure consistent and unambiguous semantic representation in glyco(proteo)mics workflows. While most human annotators would likely select the CHMO term for concepts such as SPE, it is not inherently *incorrect* to use equivalent terms from other ontologies. The choice of ontology depends not only on technical accuracy but also on community standards and interoperability goals. In this context, initiatives like MIRAGE play a key role by recommending preferred ontologies for glycomics data reporting, while still allowing for the use of alternative terms from other ontologies or controlled vocabularies when suitable concepts are not available within the recommended ontologies. A more ambitious and long-term solution would involve the development of a composite or domain-specific ontology, constructed by systematically importing and harmonizing terms from existing ontological frameworks. Such an approach could offer both semantic precision and flexibility, ensuring that domain-specific workflows in glyco(proteo)mics are accurately and consistently represented.

Some fundamental concepts such as *N-glycan release*, *O-glycan release* or *glycan purification* are not (yet) found in any ontology. Sometimes this can be circumvented with existing terms, for instance, a more specific term *PNGase F digestion* [CHMO:0002921] can be used where applicable, but more often broad terms, such as *reductive elimination* [REX:0000436] for *O-glycan release* or *Purification* [NCIT:C68780] to describe glycan purification, has been used. Some of these challenges may come from our choice of relatively recent articles, some published only a year before the time of annotation. Naturally, any new concepts or techniques introduced around this time may not yet be defined in any of the ontologies. However, in most cases, the annotators were able to find a term that accurately describes each step in the method, material or data flow. We do not expect glycomics to be unusual in this regard, such ontology gaps are common in other fields as well, including rapidly developing fields such as proteomics and machine learning. The difficulty in annotating *O*-glycan-specific methods stem from the fact that *O*-glycans are studied less frequently than *N*-glycans, approximately by a factor of two, partly due to the lack of generic enzymes. However, with increasing attention to *O*-glycans and the emergence of new analytical techniques such as *O*-glycan release by *O*-glycoproteases (Vainauskas et al. 2022), there is a pressing need to expand existing ontologies to include dedicated terms that accurately reflect these evolving workflows.

### Limitations of current Glycoscience ontologies

While ontologies more specific for glycoscience such as GLYCO and GLYCORDF (Ranzinger et al. 2015) exist, these are either obsolete (GLYCO) or were found to be too limited in coverage (GLYCORDF) to be useful in the study described here. The larger numbers of nodes and edges per word of the text suggests descriptions of methods in glyco(proteo)mics are relatively more condensed than methods in *Analytical Chemistry*, with 70 words/node and 57 words/edge compared to 90 words/node and 72 words/edge. Presumably, the reason for this is that many methods are commonplace and overlapping in glyco(proteo)mics, whereas many of the EACH100 papers introduce novel methods. Admittedly, less severe than when looking in this single journal, there is still a geographic bias in the papers’ selection that allows text mining, which likely translates into bias in both research topics and methods. Countries like Australia, the Netherlands, and Canada strongly encourage open-access publishing, with article processing charges covered by national agreements. For example, researchers in Australia can currently publish open access in most ACS, Elsevier, Springer Nature, Taylor & Francis, and Wiley journals through agreements between these publishers and the Council of Australian University Librarians. Dutch university libraries and The Royal Library of the Netherlands have similar agreements with these and other publishers, leading to 89% of the research output from academics in the Netherlands published open access in 2022, according to a 2023 report by van der Hagen and Pijpers. Topic bias is introduced by differences in disease prevalence and healthcare policies between countries, while methodological bias could be a function of the relative market shares of companies producing a particular type of technology. For example, MS vendors generally have a significantly higher market share in their home market. Awareness of this bias is important when drawing general conclusions from text-mining full-text papers.

### Pathways to improve annotation and metadata standards

To further improve the annotations, it is essential to address the absence of key glycomics-related terms in existing ontologies. However, the field of analytical chemistry is rapidly advancing, with new sample procedures and analytical platforms being developed and published nearly every day. As a result, a practical strategy is needed to support the systematic addition of new terms into relevant ontologies, while minimizing redundancy and ensuring alignment with existing entries. One straightforward approach to expand ontological coverage could involve collaborating with authors, who develop new technologies and methodologies. While promising, this strategy is likely to be time-consuming and authors may not perceive an immediate benefit in contributing formal definitions of their new innovations to ontologies. To encourage partcipation, journals could recommend, or even require, that experimental metadatabe submitted in a computer interpretable, semantic format, such as Sample and Data Relationship Format (SDRF) (Dai et al. 2021). Such policies would directly support the objectives of the MIRAGE Commission, which advocates for standardized and comprehensive reporting in glycomics. These minimum information guidelines ensure that essential experimental details, including sample preparation (Struwe et al. 2016), LC (Campbell et al. 2019), CE (Lageveen-Kammeijer et al. 2022), and MS (Kolarich et al. 2013) analyses, are thoroughly reported in publications. Notably, a dedicated workgroup within the MIRAGE initiative is currently focused on expanding ontology support for glycobiology, with active development hosted at https://gitlab.com/m708/mirage-ontologies. Beyond MIRAGE, broader glycoinformatic initiatives such as GlyGen (York et al. 2020) and the GlySpace Alliance (Lisacek et al. 2023), can play important roles in building and maintaining critical ontological infrastructure. While these initiatives are not methodology-focused per se, they bring together diverse stakeholders, including those who could contribute to a dedicated glycan-methodology ontology. Leading to improved consistency, comparability, and usability of glycomics data by supporting standardized data practices across the community. In this context, the findings of this study, particularly our identification of annotation gaps, term redundancy, and workflow complexity, could serve as a foundation for such an effort. To prioritize ontology development in a way that aligns with the community needs, one option is to consider citation counts or attention metrics to guide term addition. However, we also recognize that this approach could introduce biases and must be balanced with expert curation.

### Toward FAIR and scalable Glycoinformatics

Future efforts include a larger annotation exercise with a more diverse group of glyco(proteo)mics experts. These efforts would be greatly augmented by NLP tools and ontology mapping software capable of generating and extracting preliminary annotation. Manual curation could be further facilitated by tools that allow users to tag text in a PDF or XML version of the articles, mapping these tags to nodes and edges in a graph using terms from any ontology, and specifying the order of the steps (information that is not always evident from the order in the text). However, to our knowledge, no single tool currently offers this functionality in one integrated platform. This highlights a broader need for collaboration between experimentalists, ontologists, and tool developers. The current study contributes by identifying semantic gaps, redundancy, and complexity patterns in glyco(proteo)mics workflows, providing a foundation for the development of domain-specific ontologies and metadata standards. These improvements will not only benefit computational efforts, but also serve experimental glycobiologists by enabling more consistent, interpretable, and reusable datasets. In this way, our work supports the overarching goal of building a more interoperable and FAIR glycoscience ecosystem, aligned with the needs of both the bench and the database.

## Materials and methods

### Corpus

The selection of papers was made from a larger corpus consisting of all open-access articles on glycomics and glycoproteomics published 2020–2024 returned by a Europe PMC search query “(FIRST_PDATE:[2020-01-01 TO 2024-12-31]) AND (glycomics OR glycoproteomics) AND (OPEN_ACCESS:y)”. Open access here means “free to read and use”, which explicitly allows text mining and publishing the results of efforts such as these annotations. This search, when conducted on Jan 20, 2025, returned 2756 articles, 468 from 2020, 605 from 2021, 624 from 2022, 537 from 2023 and 522 from 2024. The retrieval and downloading of metadata and full texts in XML format were done in R using the europepmc package version 0.4.3. An analysis of the authors’ affiliations was conducted to check for geographical bias and compare this bias with that of the EACH100 set of publications. The experimental sections were extracted with XPath, and word counts were calculated using the ngram package version 3.2.2. The curators worked from the PDF versions of the articles.

### Annotations

As in the EACH100 project, a joint curation exercise was first conducted to harmonize the process. Initial structure and term mapping work was here carried out by W. W. and V. K. (at the time both last-year PhD students in the Glycomics group of the Center for Proteomics and Metabolomics). The methods in the articles were annotated as semantic directed acyclic graphs (DAGs) using GML, exactly as in the EACH100 project. In these graphs, nodes represented transformative or generative steps as identified by the curators. These nodes include changes to samples, analytes, or data, or data generation from a sample. Directed graphs were selected to clearly delineate the sequence of steps, an essential aspect for understanding the methodology. The GML format, arguably both human- and machine-readable, was chosen for its simplicity, compatibility with Cytoscape and convertibility to RDF via igraph. Curators were instructed to build a DAG for each paper’s experimental methodology, labeling nodes and edges with terms from the Ontology Lookup Service (OLS) version 4. They were advised to choose terms that most precisely represented the identified steps, but without respect to ontology. To the largest extent possible, the curators used action nouns for labeling, e.g. “*nuclear magnetic resonance spectrometry*” [CHMO:0000591] rather than “*nuclear magnetic resonance spectrometer*” [CHMO:0001807]. Issues were discussed and resolved during the annotation effort, and challenges and observations particularly relevant to glycomics and glycoproteomics were noted down in the GML files. All annotations were subsequently checked by M. P. and G. L.-K., who have more experience in semantic annotation, MS and glycomics expertise respectively.

### GML file preparation and visualization

To ensure compatibility with Cytoscape, the GML files were modified so that node labels were unique, achieved by combining the article’s (unique) DOI with the node number in each graph using R 4.4.2 with igraph 1.2.7. Cytoscape (version 3.10.3) facilitated the visualization, inspection, and merging of annotations, controlled from R using the RCy3 plugin (version 2.26.0).

## Supplementary Material

Supplemental_Information_cwaf072

## Data Availability

All R scripts, GML files, and the resulting Cytoscape session file are publicly available on GitHub: https://github.com/magnuspalmblad/GLYCO.

## References

[ref1] Alvarez MRS et al. 2022. N-glycan and glycopeptide serum biomarkers in Philippine lung cancer patients identified using liquid chromatography–tandem mass spectrometry. ACS Omega. 7:40230–40240. 10.1021/acsomega.2c05111. 36385894 PMC9647785

[ref2] Bachman JA, Gyori BM, Sorger PK. 2023. Automated assembly of molecular mechanisms at scale from text mining and curated databases. Mol Syst Biol. 19:e11325. 10.15252/msb.202211325. 36938926 PMC10167483

[ref3] Bandrowski A et al. 2016. The ontology for biomedical investigations. PLoS One. 11:e0154556. 10.1371/journal.pone.0154556. 27128319 PMC4851331

[ref4] Black M, Lamothe L, Eldakroury H et al. 2022. EDAM: the bioscientific data analysis ontology (update 2021) [version 1; not peer reviewed]. 11(ISCB Comm J):1 (poster). 10.7490/f1000research.1118900.1.

[ref5] Campbell MP et al. 2019. The minimum information required for a glycomics experiment (MIRAGE) project: LC guidelines. Glycobiology. 29:349–354. 10.1093/glycob/cwz009. 30778580

[ref6] Cao L et al. 2022. Characterization of core fucosylation via sequential enzymatic treatments of intact glycopeptides and mass spectrometry analysis. Nat Commun. 13:3910. 10.1038/s41467-022-31472-4. 35798744 PMC9262967

[ref7] Chia S et al. 2022. An integrative glycomic approach for quantitative meat species profiling. Foods. 11:1952. 10.3390/foods11131952. 35804766 PMC9265272

[ref8] Côté RG, Jones P, Martens L, Apweiler R, Hermjakob H. 2008. The ontology lookup service: more data and better tools for controlled vocabulary queries. Nucleic Acids Res. 36:W372–W376. 10.1093/nar/gkn252. 18467421 PMC2447739

[ref9] Dai C et al. 2021. A proteomics sample metadata representation for multiomics integration and big data analysis. Nat Commun. 12:5854. 10.1038/s41467-021-26111-3. 34615866 PMC8494749

[ref10] De Haan N et al. 2022. In-depth profiling of O-glycan isomers in human cells using C18 nanoliquid chromatography–mass spectrometry and glycogenomics. Anal Chem. 94:4343–4351. 10.1021/acs.analchem.1c05068. 35245040 PMC8928149

[ref11] Deutsch EW et al. 2023. Proteomics standards initiative at twenty years: current activities and future work. J Proteome Res. 22:287–301. 10.1021/acs.jproteome.2c00637. 36626722 PMC9903322

[ref12] Duivelshof BL et al. 2021. Quantitative N-glycan profiling of therapeutic monoclonal antibodies performed by middleup level HILIC-HRMS analysis. Pharmaceutics. 13:1744. 10.3390/pharmaceutics13111744. 34834160 PMC8617915

[ref13] Dutt M et al. 2023. Discovery and validation of serum glycoprotein biomarkers for high grade serous ovarian cancer. PROTEO Clin Appl. 17:e2200114. 10.1002/prca.202200114. PMC7615076 37147936

[ref14] Falck D, Wuhrer M. 2024. GlYcoLISA: antigen-specific and subclass-specific IgG fc glycosylation analysis based on an immunosorbent assay with an LC–MS readout. Nat Protoc. 19:1887–1909. 10.1038/s41596-024-00963-7. 38383719

[ref15] Hargett AA et al. 2021. Glycosylation states on intact proteins determined by NMR spectroscopy. Molecules. 26:4308. 10.3390/molecules26144308. 34299586 PMC8303171

[ref16] Hayes CA et al. 2011. UniCarb-DB: a database resource for glycomic discovery. Bioinform (Oxford, England). 27:1343. 10.1093/bioinformatics/btr137. 21398669

[ref17] Kaji H, Yamauchi Y, Takahashi N, Isobe T. 2006. Mass spectrometric identification of N-linked glycopeptides using lectin-mediated affinity capture and glycosylation site–specific stable isotope tagging. Nat Protoc. 1:3019–3027. 10.1038/nprot.2006.444. 17406563

[ref18] Kim YJ, Zaidi-Ainouch Z, Gallien S, Domon B. 2012. Mass spectrometry–based detection and quantification of plasma glycoproteins using selective reaction monitoring. Nat Protoc. 7:859–871. 10.1038/nprot.2012.023. 22498706

[ref19] Kolarich D, Jensen PH, Altmann F, Packer NH. 2012. Determination of site-specific glycan heterogeneity on glycoproteins. Nat Protoc. 7:1285–1298. 10.1038/nprot.2012.062. 22678432

[ref20] Kolarich D et al. 2013. The minimum information required for a glycomics experiment (MIRAGE) project: improving the standards for reporting mass-spectrometry-based glycoanalytic data. Mol Cell Proteomics. 12:991–995. 10.1074/mcp.O112.026492. 23378518 PMC3617344

[ref21] Lageveen-Kammeijer GSM et al. 2022. The minimum information required for a glycomics experiment (MIRAGE): reporting guidelines for capillary electrophoresis. Glycobiology. 32:580–587. 10.1093/glycob/cwac021. 35348694

[ref22] Liew CY et al. 2021. Structural identification of N-glycan isomers using logically derived sequence tandem mass spectrometry. Commun Chem. 4:92. 10.1038/s42004-021-00532-z. 36697781 PMC9814355

[ref23] Lisacek F, Tiemeyer M, Mazumder R, Aoki-Kinoshita KF. 2023. Worldwide Glycoscience informatics infrastructure: the GlySpace alliance. JACS Au. 3:4–12. 10.1021/jacsau.2c00477. 36711080 PMC9875223

[ref24] Liu J et al. 2023. Detection of N-glycoprotein associated with IgA nephropathy in urine as a potential diagnostic biomarker using glycosylated proteomic analysis. Exp Ther Med. 26:1–14. 10.3892/etm.2023.12177. PMC10518647 37753295

[ref25] Lohia S et al. 2023. Glycosylation analysis of urinary peptidome highlights IGF2 glycopeptides in association with CKD. Int J Mol Sci. 24:5402. 10.3390/ijms24065402. 36982475 PMC10048973

[ref26] Luo M et al. 2022. Site-specific N-glycosylation characterization of micro monoclonal immunoglobulins based on EThcD-sceHCDMS/MS. Front Immunol. 13:1013990. 10.3389/fimmu.2022.1013990. 36189210 PMC9520751

[ref27] Madunić K et al. 2021. Colorectal cancer cell lines show striking diversity of their O-glycome reflecting the cellular differentiation phenotype. Cell Mol Life Sci. 78:337–350. 10.1007/s00018-020-03504-z. 32236654 PMC7867528

[ref28] Nishihara S, Angata K, Aoki-Kinoshita KF, Hirabayashi J, editors. 2021. Glycoscience protocols (GlycoPODv2). Japan Consortium for Glycobiology and Glycotechnology, Tokyo, Japan. 37590565

[ref29] Pachl C, Frank N, Breitbart J, Bräse S. 2020. Overview of chemical ontologies, arXiv:2002.03842. 10.48550/arXiv.2002.03842.

[ref30] Palmblad M et al. 2022. Semantic annotation of experimental methods in analytical chemistry. Anal Chem. 94:15464–15471. 10.1021/acs.analchem.2c03565. 36281827 PMC9647698

[ref31] Parsons LM et al. 2023. Glycosylation of H4 influenza strains with pandemic potential and susceptibilities to lung surfactant SP-D. Front Mol Biosci. 10:1207670. 10.3389/fmolb.2023.1207670. 37383151 PMC10296771

[ref32] Perez-Riverol Y et al. 2022. The PRIDE database resources in 2022: a hub for mass spectrometrybased proteomics evidences. Nucleic Acids Res. 50:D543–D552. 10.1093/nar/gkab1038. 34723319 PMC8728295

[ref33] Petralia LM et al. 2022. Alteration of rhesus macaque serum N-glycome during infection with the human parasitic filarial nematode Brugia malayi. Sci Rep. 12:15763. 36131114 10.1038/s41598-022-19964-1 PMC9491660

[ref34] Polasky DA, Geiszler DJ, Yu F, Nesvizhskii AI. 2022. Multiattribute glycan identification and FDR control for glycoproteomics. Mol Cell Proteomics. 21:100205. 10.1016/j.mcpro.2022.100205. 35091091 PMC8933705

[ref35] Rabinovich GA, Van Kooyk Y, Cobb BA. 2012. Glycobiology of immune responses. Ann N Y Acad Sci. 1253:1–15. 10.1111/j.1749-6632.2012.06492.x. 22524422 PMC3884643

[ref36] Ranzinger R et al. 2015. GlycoRDF: an ontology to standardize glycomics data in RDF. Bioinform (Oxford, England). 31:919–925. 10.1093/bioinformatics/btu732. PMC4380026 25388145

[ref37] Ret D et al. 2022. DMTMMmediated methylamidation for MALDI mass spectrometry analysis of N-glycans with structurally conserved sialic acid residues in biological fluids “via direttissima”. Talanta. 242:123326. 10.1016/j.talanta.2022.123326. 35193013

[ref38] Rojas-Macias MA et al. 2019. Towards a standardized bioinformatics infrastructure for N- and O-glycomics. Nat Commun. 10:3275. 10.1038/s41467-019-11131-x. 31332201 PMC6796180

[ref39] Simon E, Swanson K, Zou J. 2024. Language models for biological research: a primer. Nat Methods. 21:1422–1429. 10.1038/s41592-024-02354-y. 39122951

[ref40] Smith B et al. 2007. The OBO foundry: coordinated evolution of ontologies to support biomedical data integration. Nat Biotechnol. 25:1251–1255. 10.1038/nbt1346. 17989687 PMC2814061

[ref41] Struwe WB et al. 2016. The minimum information required for a glycomics experiment (MIRAGE) project: sample preparation guidelines for reliable reporting of glycomics datasets. Glycobiology. 26:907–910. 10.1093/glycob/cww082. 27654115 PMC5045532

[ref42] Takakura D et al. 2023. Targeted O-glycoproteomics for the development of diagnostic markers for advanced colorectal cancer. Front Oncol. 13:1104936. 10.3389/fonc.2023.1104936. 36845686 PMC9948623

[ref43] Taylor CF et al. 2007. The minimum information about a proteomics experiment (MIAPE). Nat Biotechnol. 25:887–893. 10.1038/nbt1329. 17687369

[ref44] Tiemeyer M et al. 2017. GlyTouCan: an accessible glycan structure repository. Glycobiology. 27:915–919. 10.1093/glycob/cwx066. 28922742 PMC5881658

[ref45] Vainauskas S et al. 2022. A BroadSpecificity O-Glycoprotease that enables improved analysis of glycoproteins and glycopeptides containing intact complex O-glycans. Anal Chem. 94:1060–1069. 10.1021/acs.analchem.1c04055. 34962767

[ref46] Varki A et al. 2015. Symbol nomenclature for graphical representations of glycans. Glycobiology. 25:1323–1324. 10.1093/glycob/cwv091. 26543186 PMC4643639

[ref47] Varki A, Cummings RD, Esko JD, et al., editors. 2022. Essentials of glycobiology. 4th. Cold Spring Harbor Laboratory Press, Woodbury NY. 20301239

[ref48] Wang H et al. 2011. Integrated mass spectrometry–based analysis of plasma glycoproteins and their glycan modifications. Nat Protoc. 6:253–269. 10.1038/nprot.2010.176. 21372808 PMC4677799

[ref49] Watanabe Y, Aoki-Kinoshita KF, Ishihama Y, Okuda S. 2021. GlycoPOST realizes FAIR principles for glycomics mass spectrometry data. Nucleic Acids Res. 49:D1523–D1528. 10.1093/nar/gkaa1012. 33174597 PMC7778884

[ref50] White MEH et al. 2024. Oxonium ion scanning mass spectrometry for large-scale plasma glycoproteomics. Nat Biomed Eng. 8:233–247. 37474612 10.1038/s41551-023-01067-5 PMC10963274

[ref51] Wilkinson MD et al. 2016. The FAIR guiding principles for scientific data management and stewardship. Sci Data. 3:160018. 10.1038/sdata.2016.18. 26978244 PMC4792175

[ref52] Wilkinson H et al. 2021. The O-glycome of human nigrostriatal tissue and its alteration in Parkinson’s disease. J Proteome Res. 20:3913–3924. 10.1021/acs.jproteome.1c00219. 34191522 PMC8353623

[ref53] York WS et al. 2020. GlyGen: computational and informatics resources for glycoscience. Glycobiology. 30:72–73. 10.1093/glycob/cwz080. 31616925 PMC7335483

[ref54] Young T, Hazarika D, Poria S, Cambria E. 2018. Recent trends in deep learning based natural language processing. Eng IEEE Comput Intell Mag. 13:55–75. 10.1109/MCI.2018.2840738.

[ref55] Zhou Y et al. 2023. Thiol-ene-based microfluidic chips for glycopeptide enrichment and online digestion of inflammation-related proteins osteopontin and immunoglobulin G. Anal Bioanal Chem. 415:1173–1185. 10.1007/s00216-022-04498-2. 36607393 PMC9817458

