# Supplementary material for: Semantic annotation of Glycomics and Glycoproteomics methods
Source: Glycobiology. 2025 Oct 31;36(5):cwaf072. doi: 10.1093/glycob/cwaf072 (PMC13019685; doi:10.1093/glycob/cwaf072)
Supplement: Supplemental_Information_cwaf072 [file supplemental_information_cwaf072.pdf]

List of Tables

S1 Additional information on the 20 annotated papers. 2
S2 The ontologies and their frequencies in node, edge and paper annotations. 3

Table S1: Additional information on the 20 annotated papers.

Author	DOI	Primary method(s)	Type of study/application	Used ontologies	PMCID
Duivelshof	10.3390/pharmaceutics13111744	HILIC-FLD-HRMS	profiling	CHMO,ERO,REX,MOP,NCIT	PMC8617915
Hargett	10.3390/molecules26144308	NMR	RNAse B	CHMO,ERO,MS,NCIT,NMR	PMC8303171
Liew	10.1038/s42004-021-00532-z	LODES/MSn	isomer seperation	CHMO,OBI,NCIT,NMR	PMC9814355
Madunić	10.1007/s00018-020-03504-z	PGC, nano-LC–ESI-MS/MS	small cancer cohort	CHMO,EDAM,NCIT,OBCS,OBI,OMIT,REX	PMC7867528
Ret	10.1016/j.talanta.2022.123326	MALDI	characterization of N-glycans	CHMO,EDAM,OBI,OMIT,SIO	[1]
Wilkinson	10.1021/acs.jproteome.1c00219	WAX, HILIC, PGC, LC-MS/MS, exoglucosidase arrays	Parkinson's Disease	CHMO,EDAM,NCIT,OBI,REX	PMC8353623
Alvarez	10.1021/acsomega.2c05111	LC-MS/MS	Philippine Lung Cancer	CHMO,EDAM,NCIT,OBCS,OBI,OMIT	PMC9647785
Chia	10.3390/foods11131952	LC-MS (TOF)	food analysis	CHMO,EDAM,ERO,NCIT,OBI,OMIT	PMC9265272
Gao	10.1038/s41467-022-31472-4	LC-MS/MS	characterization of core fucosylation	CHMO,EDAM,ERO,GO,NCIT,OBI,REX	PMC9262967
Luo	10.3389/fimmu.2022.1013990	EThcD-sceHCD-MS/MS	N-glycosylation characterization	CHMO,EDAM,GO,NCIT,OBI,REX	PMC9520751
Petralia	10.1038/s41598-022-19964-1	MALDI-TOF and HILIC LC-MS	parasitology	AFP,CHMO,EDAM,MI,NCIT,OBCS,OBI,OMIT	PMC9491660
Polasky	10.1016/j.mcpro.2022.100205	bioinformatics	glycopeptide annotation	EDAM,OMIT	PMC8933705
de Haan	10.1021/acs.analchem.1c05068	LC-MS	isomer separation, new O-glycan release method	CHMO,NCIT,OBI,	PMC8928149
Dutt	10.1002/prca.202200114	LC-MS/MS	high grade serous ovarian cancer	CHMO,EDAM,OBI,GO,GSSO,MOD,MOP,NCIT	PMC7615076
Liu	10.3892/etm.2023.12177	LC-MS/MS	IgA nephropathy in urine	CHMO,ERO,GO,GSSO,MMO,MOD,MOP,NCIT,OBI	PMC10518647
Lohia	10.3390/ijms24065402	CE-MS/MS	CKD	CHMO,EDAM,GSSO,NCIT,OBI	PMC10048973
Parsons	10.3389/fmolb.2023.1207670	nanoLC-MS and MALDI-TOF	Glycosylation of H4 influenza strains	CHMO,GO,MI,NCIT,OBI,OMIT,REPR,REX,SIO	PMC10296771
Takakura	10.3389/fonc.2023.1104936	LC-MS/MS	colorectal cancer	CHMO,EDAM,FBcv,NCIT,OBI	PMC9948623
Zhou	10.1007/s00216-022-04498-2	microfluidics	glycoproteins	CHMO,EDAM,NCIT,OMIT,REPR	PMC9817458
White	10.1038/s41551-023-01067-5	LC-MS/MS	Profiling,COVID-19,glycopeptides quantification	CHMO,EDAM,EFO,END,MS,NCIT,OBI,OMIT	PMC10963274

Note: [[1]] This paper is open access, but not in PubMed Central and hence lacks PMC identifier.

Table S2: The ontologies and their frequencies in node, edge and paper annotations.

Ontology	Title	Ontology IRI	Nodes	Edges	Papers
AFO	Allotrope Foundation Ontology Merged Without QUDT And Inferred	http://purl.allotrope.org/voc/afo/latest.xml	1	1	1
BTO	The BRENDA Tissue Ontology	http://purl.obolibrary.org/obo/bto.owl	-	8	-
CHEBI	Chemical Entities of Biological Interest	http://purl.obolibrary.org/obo/chebi.owl	-	179	-
CHEMINF	chemical information ontology	http://semanticchemistry.github.io/semanticchemistry/ontology/cheminf.owl	-	2	-
CHMO	Chemical Methods Ontology	http://purl.obolibrary.org/obo/chmo.owl	169	15	19
EDAM	Bioinformatics operations, data types, formats, identifiers and topics	http://edamontology.org/EDAM.owl	47[1]	161[2]	15
EFO	Experimental Factor Ontology	http://www.ebi.ac.uk/efo/efo.owl	1	-	1
EFO	Experimental Factor Ontology	http://www.ebi.ac.uk/efo/efo.owl	1	-	1
ERO	eagle-i resource ontology	http://purl.obolibrary.org/obo/ero.owl	7	-	6
FBCV	FlyBase Controlled Vocabulary	http://purl.obolibrary.org/obo/fbcv.owl	2	-	1
GO	Gene Ontology	http://purl.obolibrary.org/obo/go/extensions/go-plus.owl	6	-	5
GSSO	the Gender, Sex, and Sexual Orientation ontology	http://purl.obolibrary.org/obo/gsso.owl	5	-	3
MI	Molecular Interactions Controlled Vocabulary	http://purl.obolibrary.org/obo/mi.owl	2	-	2
MMO	Measurement method ontology	http://purl.obolibrary.org/obo/mmo.owl	1	-	1
MOD	Protein modification	http://purl.obolibrary.org/obo/mod.owl	2	-	2
MOP	molecular process ontology.	http://purl.obolibrary.org/obo/mop.owl	3	-	3
MS	Mass spectrometry ontology	http://purl.obolibrary.org/obo/ms.owl	3	1	2
MSIO	Metabolomics Standards Initiative Ontology	http://purl.obolibrary.org/obo/msio.owl	-	12	-
NCIT	NCI Thesaurus OBO Edition	http://purl.obolibrary.org/obo/ncit.owl	136	114	18
NMR	nuclear magnetic resonance CV	http://nmrML.org/nmrCV	2	-	2
OBCS	Ontology of Biological and Clinical Statistics	http://purl.obolibrary.org/obo/obcs.owl	3	-	3
OBI	Ontology for Biomedical Investigations	http://purl.obolibrary.org/obo/obi.owl	46	5	15
OMIT	Ontology for MIRNA Target	http://purl.obolibrary.org/obo/omit.owl	22	52	10
PR	PRotein Ontology	http://purl.obolibrary.org/obo/pr.owl	-	17	-
REPR	REPRODUCE-ME Ontology	https://w3id.org/reproduceme	2	-	2
REX	Physico-chemical process	http://purl.obolibrary.org/obo/rex.owl	7	-	6
SCDO	Sickle Cell Disease Ontology	http://purl.obolibrary.org/obo/scdo.owl	-	5	-
SIO	Semanticscience Integrated Ontology	http://semanticscience.org/ontology/sio.owl	3	5	2
STATO	the statistical methods ontology	http://purl.obolibrary.org/obo/stato.owl	-	1	-
UBERON	Uberon multi-species anatomy ontology	http://purl.obolibrary.org/obo/uberon.owl	-	2	-

Note: [1] These annotations are from the "operations" subontology or branch of EDAM [2] These annotations are from the "data" (type) EDAM branch
